# COVID-19-Related Web Search Behaviors and Infodemic Attitudes in Italy: Infodemiological Study

**DOI:** 10.2196/19374

**Published:** 2020-05-05

**Authors:** Alessandro Rovetta, Akshaya Srikanth Bhagavathula

**Affiliations:** 1 Mensana srls Research and Disclosure Division Brescia Italy; 2 Institute of Public Health, College of Medicine and Health Sciences United Arab Emirates University Abu Dhabi United Arab Emirates

**Keywords:** novel coronavirus, COVID-19, Google search, Google Trends, infodemiology, infodemic monikers, Italy, behavior, public health, communication, digital health, online search

## Abstract

**Background:**

Since the beginning of the novel coronavirus disease (COVID-19) outbreak, fake news and misleading information have circulated worldwide, which can profoundly affect public health communication.

**Objective:**

We investigated online search behavior related to the COVID-19 outbreak and the attitudes of “infodemic monikers” (ie, erroneous information that gives rise to interpretative mistakes, fake news, episodes of racism, etc) circulating in Italy.

**Methods:**

By using Google Trends to explore the internet search activity related to COVID-19 from January to March 2020, article titles from the most read newspapers and government websites were mined to investigate the attitudes of infodemic monikers circulating across various regions and cities in Italy. Search volume values and average peak comparison (APC) values were used to analyze the results.

**Results:**

Keywords such as “novel coronavirus,” “China coronavirus,” “COVID-19,” “2019-nCOV,” and “SARS-COV-2” were the top infodemic and scientific COVID-19 terms trending in Italy. The top five searches related to health were “face masks,” “amuchina” (disinfectant), “symptoms of the novel coronavirus,” “health bulletin,” and “vaccines for coronavirus.” The regions of Umbria and Basilicata recorded a high number of infodemic monikers (APC weighted total >140). Misinformation was widely circulated in the Campania region, and racism-related information was widespread in Umbria and Basilicata. These monikers were frequently searched (APC weighted total >100) in more than 10 major cities in Italy, including Rome.

**Conclusions:**

We identified a growing regional and population-level interest in COVID-19 in Italy. The majority of searches were related to amuchina, face masks, health bulletins, and COVID-19 symptoms. Since a large number of infodemic monikers were observed across Italy, we recommend that health agencies use Google Trends to predict human behavior as well as to manage misinformation circulation in Italy.

## Introduction

The internet is the largest and fastest source to obtain health information, and millions of people seek health information online every day [1]. In the context of the novel coronavirus disease (COVID-19) pandemic, people around the world are forced to stay at home and turn to the internet for work and to stay connected with others. As the COVID-19 outbreak continues, the need to obtain information about the disease, its prevention, and risk communication has become greater for people.

“Infodemiological” methods, such as an online search of traffic on Google, are widely used to understand the searching behaviors of the public during an epidemic, as well as for public health surveillance purposes [2-7]. Several online sources, such as Facebook, Twitter, and electronic health records, have wide application in infodemiological studies [8-10]. Indeed, the Google Trends tool provides both real-time and achieved information on trends (eg, variations in online interest in selected keywords and topics over time) [11-13]. In particular, Google Trends enables the analysis and forecasting of sensitive health topics such as AIDS, illegal drug use, and metal health [13]. Therefore, trend data generated by Google search volume can offer valuable insights into population behavior and health-related phenomena, particularly during infectious disease outbreaks [7,14-17]. Since the beginning of the COVID-19 outbreak, fake news and misleading information have circulated all over the world, which profoundly affect public health communication and diminish preventive measures [18-21]. In this context, we investigated online search query behavior related to this pandemic and the extent of infodemic monikers circulating in Italy.

## Methods

### Search Methodology

We used Google Trends to explore internet search activity related to COVID-19 from January 21, 2020, to March 24, 2020. Article titles from the most read national newspapers and government websites were mined to investigate the extent and attitudes of various infodemic monikers related to COVID-19 that were circulating in Italy during the study period. We defined “infodemic monikers” as information that was substantially erroneous, which gave rise to interpretative mistakes, fake news, episodes of racism, or any other form of misleading information circulating on the internet.

Google Trends is an online tool that tracks keyword search queries users input in the Google search engine and determines their popularity and volume. It provides information on the search query according to a specific time period and location. The search volume results are scaled on a range of 0 (very low) to 100 (very high). Google Trends allows for the retrieval of queries for any keyword entered; up to five groups of terms can be compared at one time to explore the online interest in each term. By using this technique, we retrieved data from Google Trends using the keywords “Coronavirus” and “Coronavirus+” in the English and Italian languages. Each query with these keywords were also researched as the “search term” and “search topic.” The “search term” provides the results for all keywords that fall within the category and the “search topic” provides the results of a group of terms that share the same concept in any language.

We used a previously described framework by Mavragani et al [22] for the region selection and time period selection to retrieve query data from Google Trends. First, we searched for the keyword COVID-19 and related terms at the country level to understand overall interest. Second, using this information, we retrieved interest by city and regions across Italy. Each keyword was searched independently between January 21, 2020, and March 24, 2020. The data showing high values were further investigated manually to identify any event linked to the top searches. These queries were also cross-checked with news bulletins. By doing so, we identified the various infodemic monikers circulating across the country.

We reviewed the headlines of newspaper articles and government reports to identify their contribution in spreading infodemic monikers to the public. In order to obtain the search information from these media outlets, we used specific keywords frequently used in news and government report titles to quantify the average information values (AVs) of terms. The AVs were calculated as the number of monikers used in the headlines per 5 days. In order to characterize the obtained infodemic monikers, we categorized infodemic attitudes into 4 groups:

1. Superficial attitude:
  the user adopts words that can generate confusion since they do not uniquely identify the topic (eg, coronavirus).
2. Misinformative attitude:
  the user adopts words that can lead to the spread of fake news (eg, 5G coronavirus).
3. Racist attitude:
  the user adopts words that, voluntarily or not, generate or accentuate episodes of racism (eg, Chinese coronavirus).
4. Definitive attitude:
  the user adopts the most appropriate terms for the correct identification of the query (eg, COVID-19).

### Available Data and Materials, Ethical Approval, and Funding

All materials were obtained from anonymous open-source data. Thus, ethical approval was not required. No external funding was provided for this study.

## Results

### Overview

**Figure 1:**
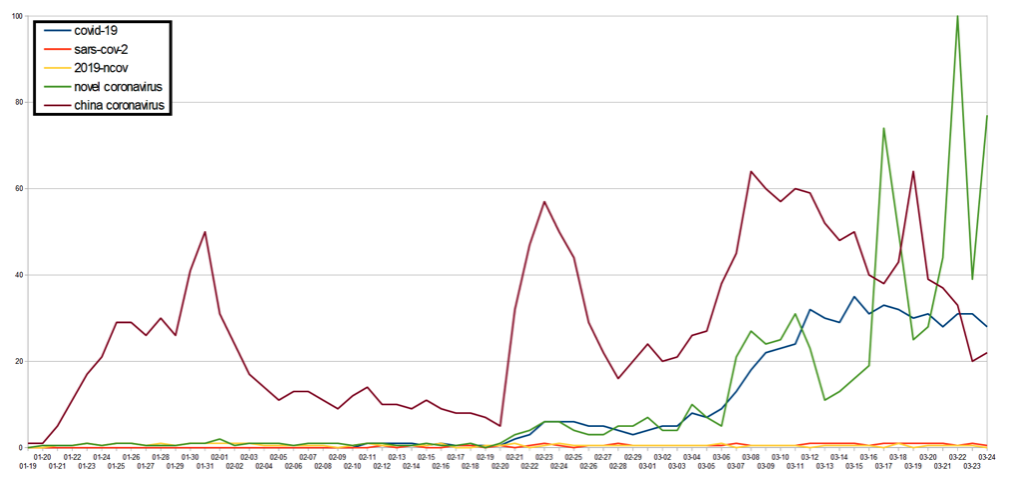
The top infodemic and scientific terms relating to coronavirus disease (COVID-19) trending in Italy.

The top five infodemic and scientific COVID-19 terms trending in Italy, according to inputs in Google search, were “novel coronavirus,” “China coronavirus,” “COVID-19,” “2019-nCOV,” and “SARS-COV-2” (Figure 1). From February 20 to March 24, 2020, the keyword that yielded the greatest search value was “coronavirus”; it had a search volume of 59 (SD 9). The other keywords’ average peak comparison (APC) values were neglected compared to the latter (Multimedia Appendix 1). The keywords that showed APC<1 are omitted for further investigation. On March 22, 2020, excluding the term “coronavirus” from the cluster, the query related to “novel coronavirus” had the highest value (ie, 100). On the previous day, Italy recorded the highest number of new cases (n=6577), and the government enforced lockdown measures. In contrast, “China coronavirus” was the most commonly used query since the beginning of the COVID-19 outbreak in January 2020. Furthermore, the terms “China coronavirus” (value 38, SD 4), “novel coronavirus” (value 21, SD 6), and “COVID-19*”* (value 17, SD 3) were the most frequently used queries since February 20, 2020, when Italy become an epicenter of the COVID-19 outbreak.

**Figure 2:**
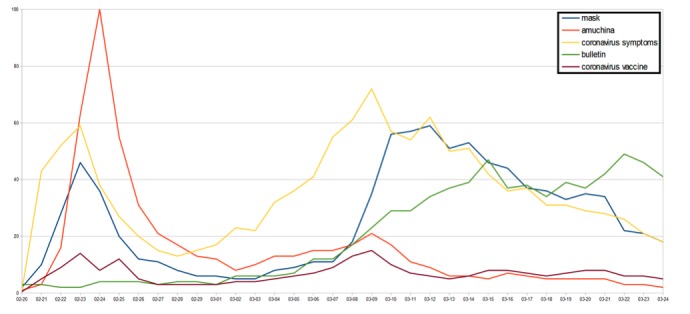
The top five searches related to health.

With respect to public restlessness in Italy, “face masks,” “amuchina” (disinfectant) (value 23, SD 6), “symptoms of the novel coronavirus,” “health bulletin,” and “vaccine for coronavirus” were the top five searches related to health. During the early period of the COVID-19 outbreak, there was a spike in queries regarding symptoms, followed by face masks and disinfectants (Figure 2). In particular, on February 22, 2020, disinfectant-related searches in Italy reached the breakout stage, with a search value of 100. Later, public restlessness appeared to drive an immense increase in queries related to the symptoms of COVID-19. Moreover, on March 11, 2020, there was a tremendous increase in the top five searches related to COVID-19.

**Figure 3:**
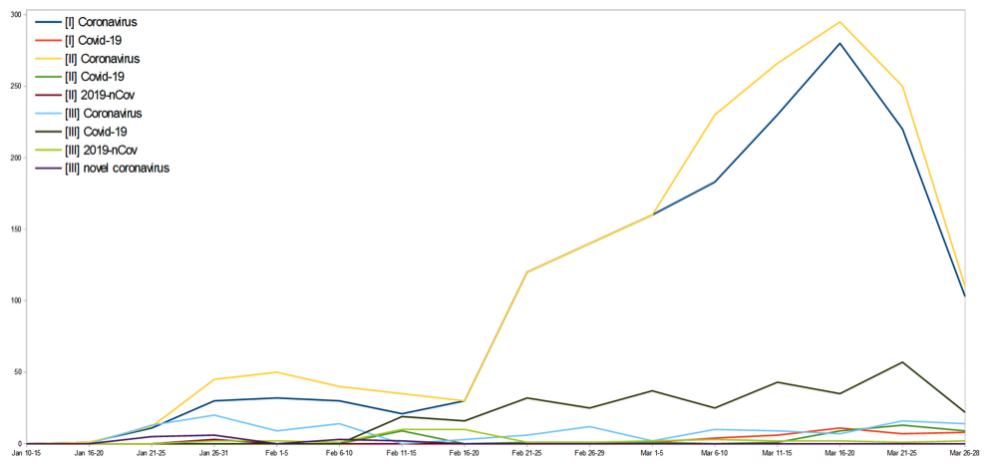
Keywords used to identify information related to coronavirus disease (COVID-19): *Il Sole 24 Ore* (I) and *La Repubblica* (II) newspapers, and government bulletins (III).

We also referred to two widely read Italian newspapers—*Il Sole 24 Ore* and *La Repubblica*—that have been publishing a large number of articles related to COVID-19, as well as government websites, to investigate AVs. We found that most of the Italian public used the keyword “coronavirus” to obtain information in *La Repubblica (*AV 127, SD 50) and *Il Sole 24 Ore* (AV 113, SD 46), while government bulletins were not routinely used (AV 22, SD 9). Detailed information on the keywords used to identify information related to COVID-19 during the pandemic period is shown in Figure 3 and Multimedia Appendix 2.

**Figure 4:**
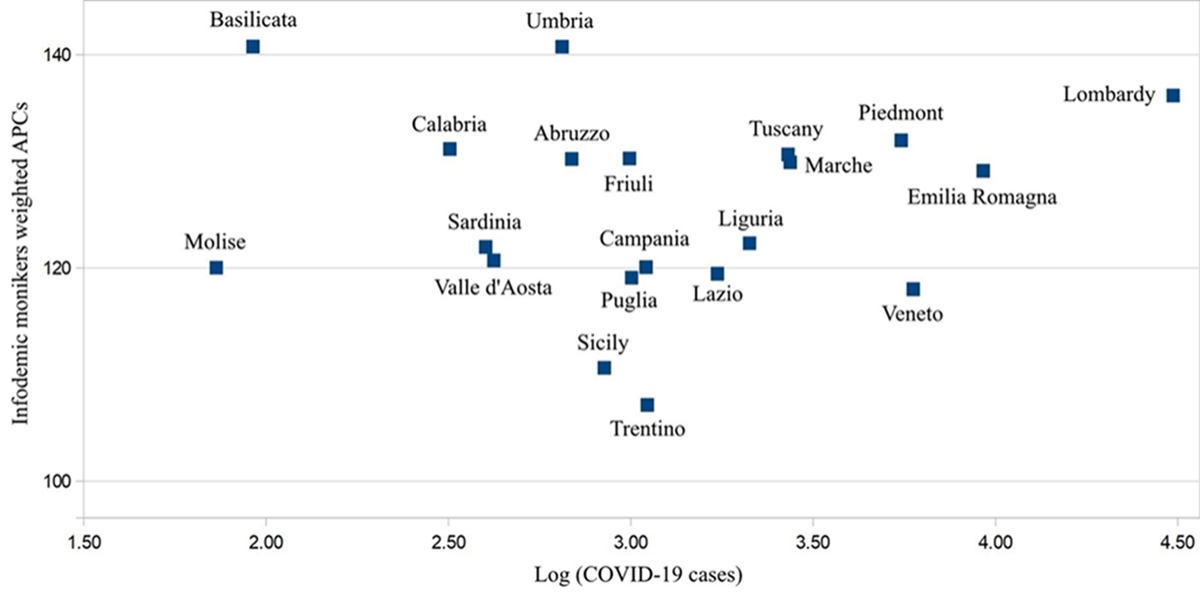
Regional dispersion of infodemic monikers about coronavirus disease (COVID-19) in Italy. APC: average peak comparison.

**Figure 5:**
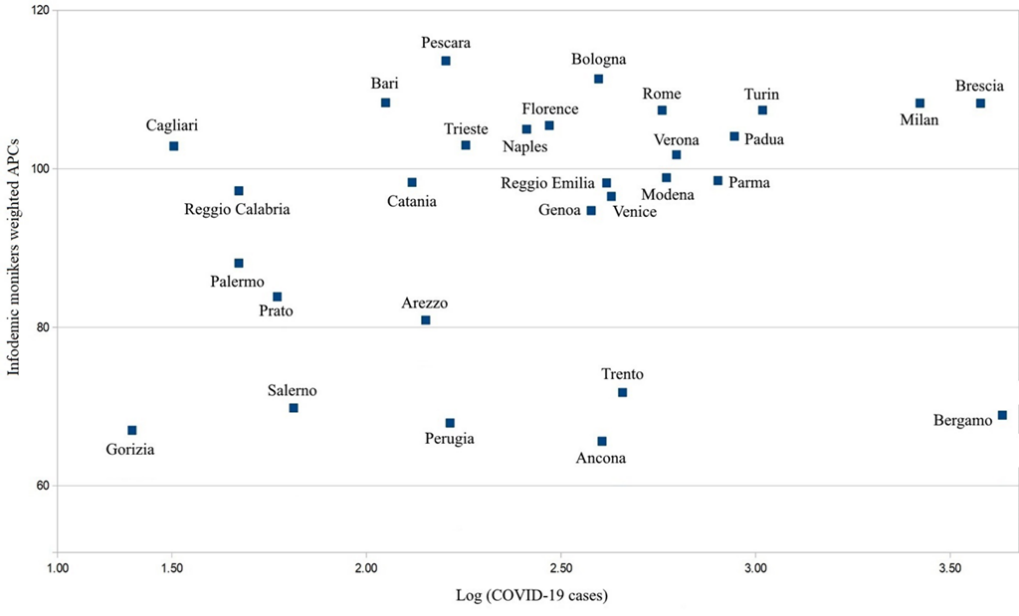
Dispersion of infodemic monikers about coronavirus disease (COVID-19) circulating across various cities in Italy. APC: average peak comparison.

Our findings indicate that the regions with the most amount of COVID-19 cases were not always the first to circulate key infodemic monikers. For instance, regions such as Umbria and Basilicata had the highest number of infodemic monikers (APC weighted total >140), while the number of cases reported in these regions was limited from January to March 2020 (Figure 4). Furthermore, the presence of these monikers was particularly pronounced (APC weighted total >100) across several cities in Italy, in particular, Pescara and Bologna (Figure 5).

### Infodemic Attitudes

The infodemic attitudes of various types of information that circulated across Italy during the study period are presented in Table 1. Most COVID-19-related information that circulated in the regions of Basilicata, Umbria, and Emilia Romagna were found to be superficial and did not provide clearer information on COVID-19. Misinformation was widespread in Umbria and Basilicata. As COVID-19 spread across the world from China, most information related to racism, such as “China coronavirus,” “Chinese virus,” “Chinese coronavirus,” and “Wuhan virus,” were more frequently searched in the Campania and Friuli Venezia Giulia regions.

**Table 1:** Attitudes of infodemic monikers on coronavirus disease (COVID-19) in circulation across Italy between January 21, 2020, and March 24, 2020.

Region	COVID-19 cases^a^, n	Total APC^b^ value	APC values of infodemic attitudes (1-100)			
			Superficial	Misinformation	Racial	Definitive
Lombardia	30,703	275	95	68	83	71
Emilia Romagna	9254	296	97	79	89	69
Veneto	5948	256	84	61	82	71
Piemonte	5515	286	96	76	89	75
Marche	2736	279	93	78	88	80
Toscana	2699	293	94	88	89	78
Liguria	2116	270	88	74	90	82
Lazio	1728	269	89	76	79	75
Campania	1101	281	88	75	100	82
Trentino-Alto Adige	1110	228	80	63	57	72
Puglia	1005	268	87	78	87	84
Friuli Venezia Giulia	992	267	94	75	98	100
Sicilia	846	268	81	68	84	65
Abruzzo	689	292	92	84	97	81
Umbria	648	312	97	100	92	77
Valle d'Aosta	400	239	89	70	36	56
Sardegna	421	255	89	64	95	93
Calabria	319	281	91	86	87	83
Basilicata	92	306	100	92	96	82
Molise	73	237	87	84	66	92

^a^Assessed between January 21, 2020, and March 24, 2020.

^b^APC: average peak comparison.

## Discussion

### Principal Findings

This is the first study to investigate the online search behaviors of the public in the context of the COVID-19 pandemic. We aimed to uncover the extent and the attitudes of infodemic monikers that circulated in Italy during the study period. Previously published studies have investigated Google Trends and Twitter activities related to COVID-19 but were conducted in China [23,24], Taiwan [25], the United States [26], and Spain [27]. In summary, we identified “novel coronavirus,” “China coronavirus,” “COVID-19,” “2019-nCOV,” and “SARS-COV-2” as the top infodemic and scientific COVID-19 terms trending in Italy. “Face masks,” “amuchina,” “symptoms of the novel coronavirus,” “health bulletins,” and “vaccines for coronavirus” were the top five searches related to health. Several infodemic monikers have widespread circulation in major Italian cities. In particularly, misinformation was widely circulated in the Campania region and racism-related information in Umbria and Basilicata.

The current COVID-19 pandemic has threatened global public health and has generated millions of internet searches worldwide. In Italy, ”China coronavirus“ was the most frequently searched term on Google, coinciding with the first incidence of COVID-19 in 2 Chinese tourists, as announced by the Italian Prime Minister Giuseppe Conte at the end of January 2020 [28]. However, the increasing number of cases did not generate a significant number of web searches until the World Health Organization (WHO) declared the COVID-19 outbreak as a pandemic [29], and the Italian government imposed draconian rules to stop the spread in early March 2020 [30]. Notably, queries related to COVID-19 symptoms, disinfectants, masks, and vaccines were relatively high in the fourth week of February 2020, stabilized in 20 values during early March, and quickly increased as the number of cases increased in Italy. This is indicative of peoples’ restlessness with regard to gathering information about necessary personal protection and hygiene practices as COVID-19 cases rose in Italy. Of note, around 40,000 people were charged for violating the lockdown, and the often-mentioned reasons to go out were ”amuchina,“ ”face masks,“ and other casual reasons [31]. These reasons are also reflected in our research and, thus, diminish countermeasures for the outbreak in Italy. To curtail this, the government has initiated a “self-certification” form to declare a valid reason such as work, health reasons, or buying food that necessitates leaving the house.

The findings of our study suggest that web search interest in COVID-19, both at the regional level and in cities in Italy, were influenced by tradition, electronic newspapers, and print media coverage. For instance, people preferred to use the term ”Coronavirus“ more frequently to obtain information in newspapers instead of “COVID-19,” “2019-nCOV,” and “novel coronavirus.” Data from previous research suggest delivering information through Twitter and electronic news outlets frequently focus more on spreading news disproportionately than awareness and educational campaigns [32-34]. These observations have important implications in generating COVID-19-related restlessness in the general public in Italy. Further research is warranted.

Through our investigation, we identified several infodemic monikers of COVID-19 that impinged public communication across various cities in Italy. Misinformation during an outbreak can profoundly affect public health communication and create xenophobia between nations [35-39]. Disseminating fake news and racism across social media has become a widespread practice, and the COVID-19 outbreak is no exception [39,40]. Misinformation and anti-Asian sentiments have increased around the world [39,41,42]. In Italy, several incidences of discrimination and anti-Chinese sentiments were reported [43-45]; however, we believe that the rate of information related to racism that circulated across the country could be the true confounding factor contributing to xenophobia.

The failures of Chinese authorities to handle the virus at an early stage has resulted in the spread of COVID-19 across the world, with new cases arising from ongoing human-to-human transmission as well as from asymptomatic individuals [46]. Additionally, preliminary investigations by the WHO denied the possibility of human-to-human transmission of COVID-19 [47]. We assume that this type of misleading information may have resulted in the instigation of angry online conversations among netizens in Italy. Although we did not delve deeper into the type of potential misinformation that spread across Italy, we believe that dispersing misinformation can create agitation, cause fear, and ultimately diminish preventive measures for the outbreak. Journalists and mass media regulators have an important role in delivering comprehensive information to citizens, as well as taking serious actions on those spreading misinformation.

### Limitations

Our study had some limitations to consider. Google Trends captures the search behavior of people who use the Google search engine. Consequently, people using other search engines were not investigated. Also, we relied on the accuracy of data provided by Google Trends and do not have any information about the methods used by Google to generate search data and algorithms.

### Conclusion

Using Google Trends, the present study identified that Google search query data reflect a growing regional and population-level interest in COVID-19. Searches related to disinfectants, face masks, health bulletins, and vaccines and symptoms related to COVID-19 were top search keywords. However, a large number (APC weighted total >140) of infodemic monikers have been circulating in Italy. Therefore, health agencies can use Google Trends to predict human behavior as well as tackle the misinformation that is currently circulating in Italy.

## Appendix

Multimedia Appendix 1 The most searched infodemic words for group type.

Multimedia Appendix 2 Keywords used to search the infodemic monikers.

## Abbreviations

APC: average peak comparison
AV: average information value
COVID-19: coronavirus disease
WHO: World Health Organization
