# Supplementary material for: COVID-19-Related Web Search Behaviors and Infodemic Attitudes in Italy: Infodemiological Study
Source: JMIR Public Health Surveill. 2020 May 5;6(2):e19374. doi: 10.2196/19374 (PMC7202310; doi:10.2196/19374)

**Multimedia Appendix 1**

We used a cluster of words that formed using the two most searched infodemic words for group type (coronavirus and china coronavirus) and the scientific denominations (COVID-19, SARS-COV-2, 2019-nCOV and novel coronavirus). The comparison was made to highlight the discrepancy between the infodemic and scientific names. Below are the top five COVID-19 queries, regardless of the group they belong to, they are: coronavirus, italy coronavirus, news coronavirus, latest (news) coronavirus, breaking news coronavirus.


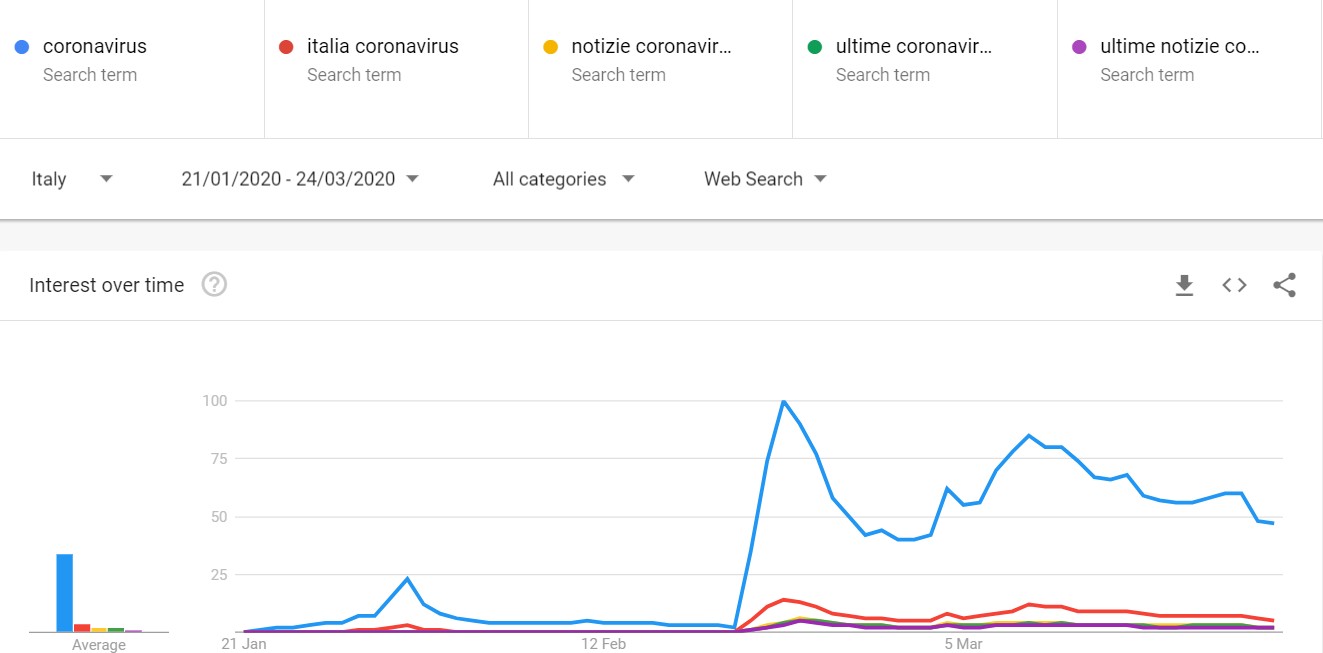


The difference in the exclusion and inclusion of coronavirus was also observed to see any differences in the interest.
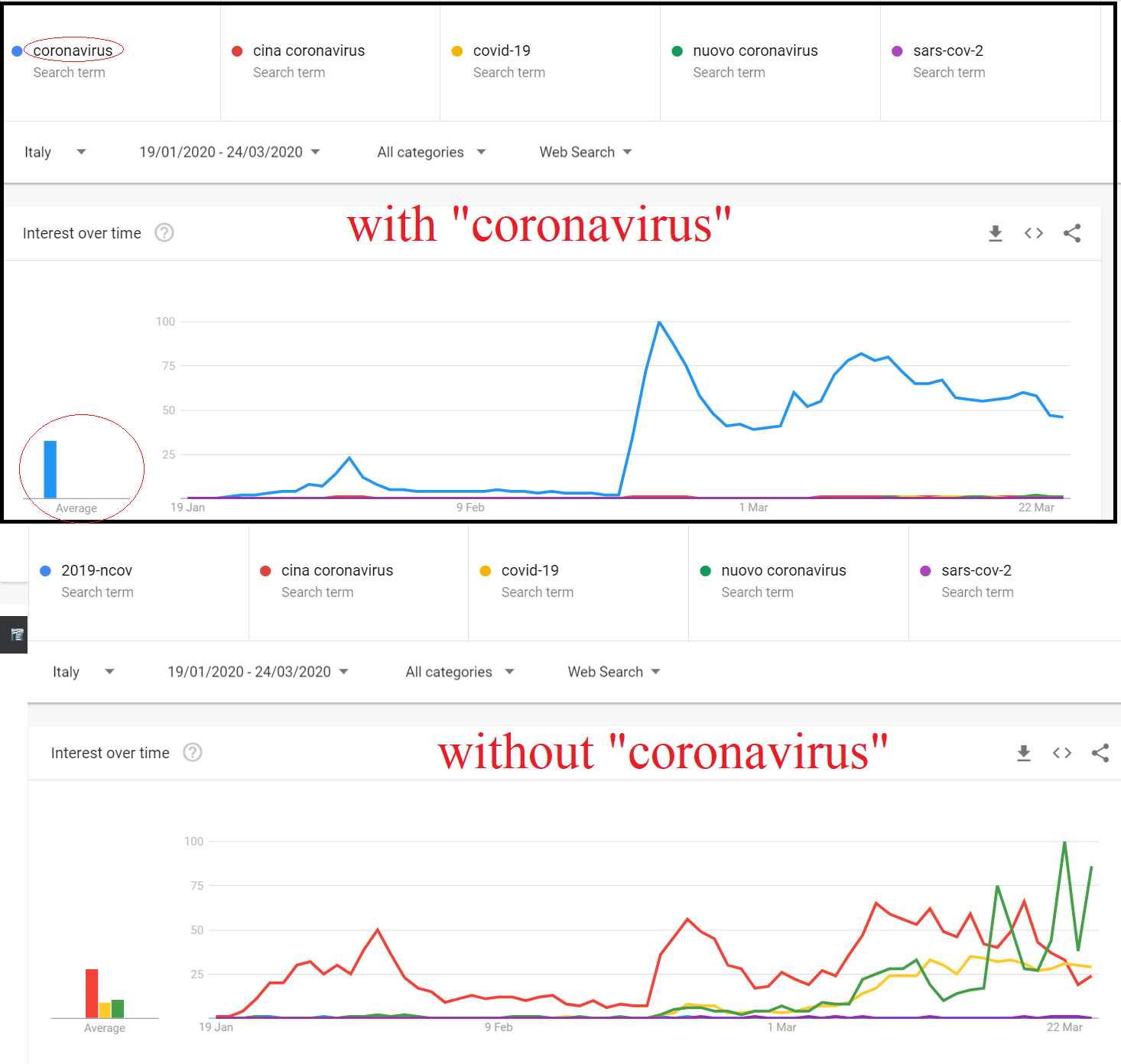

Supplement: Multimedia Appendix 1 [file publichealth_v6i2e19374_app1.docx]
