# Supplementary material for: COVID-19-Related Web Search Behaviors and Infodemic Attitudes in Italy: Infodemiological Study
Source: JMIR Public Health Surveill. 2020 May 5;6(2):e19374. doi: 10.2196/19374 (PMC7202310; doi:10.2196/19374)
Supplement: Multimedia Appendix 2 [file publichealth_v6i2e19374_app2.docx]

**Multimedia Appendix 2**

**Keywords used to search the infodemic monikers:**

**Infodemic group 1:** coronavirus, coronavirus italy, coronavirus latest news, coronavirus news, coronavirus symptoms, coronavirus lombardy, coronavirus cases, coronavirus today, coronavirus contagion, coronavirus decree.

**Infodemic group 2:** coronavirus 5G, homemade coronavirus cure, coronavirus laboratory, coronavirus conspiracy, coronavirus montanari (public figure source of fake news), coronavirus sgarbi (public figure source of fake news).

**Infodemic group 3:** china coronavirus, wuhan coronavirus, chinese coronavirus, chinese laboratory coronavirus.

**Infodemic group 4:** covid-19, sars-cov-2, ncov-2019, novel coronavirus

**Non infodemic queries:** amuchina, mask, bulletin, swab, conte conference, amuchina recipe, do-it-yourself amuchina, homemade amuchina, do-it-yourself amuchina, parchment paper mask, how to make a mask, tutorial mask, self-certification.
